# Hepatitis C bio-behavioural surveys in people who inject drugs—a systematic review of sensitivity to the theoretical assumptions of respondent driven sampling

**DOI:** 10.1186/s12954-017-0172-z

**Published:** 2017-07-11

**Authors:** Ryan Buchanan, Salim I. Khakoo, Jonathan Coad, Leonie Grellier, Julie Parkes

**Affiliations:** 1 Department of Population Science and Medical Statistics, Faculty of Medicine, University of Southampton, C level, South Academic block, Southampton, Hampshire UK; 2 Faculty of Medicine, University of Southampton, E level, South Academic block, Southampton, Hampshire UK; 3 Department of Gastroenterology, St Mary’s Hospital, Isle of Wight, Newport, UK

**Keywords:** Hepatitis C, Prevalence, Respondent-driven sampling, Intravenous, Drug

## Abstract

**Background:**

New, more effective and better-tolerated therapies for hepatitis C (HCV) have made the elimination of HCV a feasible objective. However, for this to be achieved, it is necessary to have a detailed understanding of HCV epidemiology in people who inject drugs (PWID).

Respondent-driven sampling (RDS) can provide prevalence estimates in hidden populations such as PWID. The aims of this systematic review are to identify published studies that use RDS in PWID to measure the prevalence of HCV, and compare each study against the STROBE-RDS checklist to assess their sensitivity to the theoretical assumptions underlying RDS.

**Method:**

Searches were undertaken in accordance with PRISMA systematic review guidelines. Included studies were English language publications in peer-reviewed journals, which reported the use of RDS to recruit PWID to an HCV bio-behavioural survey. Data was extracted under three headings: (1) survey overview, (2) survey outcomes, and (3) reporting against selected STROBE-RDS criteria.

**Results:**

Thirty-one studies met the inclusion criteria. They varied in scale (range 1–15 survey sites) and the sample sizes achieved (range 81–1000 per survey site) but were consistent in describing the use of standard RDS methods including: *seeds*, coupons and recruitment incentives.

Twenty-seven studies (87%) either calculated or reported the intention to calculate population prevalence estimates for HCV and two used RDS data to calculate the total population size of PWID. Detailed operational and analytical procedures and reporting against selected criteria from the STROBE-RDS checklist varied between studies. There were widespread indications that sampling did not meet the assumptions underlying RDS, which led to two studies being unable to report an estimated HCV population prevalence in at least one survey location.

**Conclusion:**

RDS can be used to estimate a population prevalence of HCV in PWID and estimate the PWID population size. Accordingly, as a single instrument, it is a useful tool for guiding HCV elimination. However, future studies should report the operational conduct of each survey in accordance with the STROBE-RDS checklist to indicate sensitivity to the theoretical assumptions underlying the method.

**Systematic review registration:**

PROSPERO CRD42015019245

## Background

Hepatitis C (HCV) infects over 150 million people and alongside other causes of viral hepatitis is a leading cause of death worldwide [1, 2]. New, more effective and better-tolerated treatments for HCV are now widely available; however, the accurate epidemiological data required to guide the logistical and financial planning needed for disease elimination strategies is lacking [3].

Over ten million people worldwide who currently inject drugs (PWID) and many more with a past history of injecting drug use are thought to be chronically infected with HCV [4], and it is the treatment of PWID rather than other patients with HCV that is likely to have the most profound impact on HCV prevalence [5]. However, it is within this population that there are particular concerns about the validity of epidemiological estimates as these are generally based on data from convenience samples in ‘easy to reach’ PWID [4, 6].

PWID are hidden by social stigma and the illegality of their practice, and therefore, it is difficult to obtain a representative sample necessary to make population prevalence estimates [7]. Interest and experience in studying hard-to-reach or hidden populations developed substantially in the wake of the HIV epidemic in the 1990s. At this time, the difficultly of obtaining representative samples with existing survey techniques prompted the development of a method called respondent-driven sampling (RDS) [8].

RDS begins with a sample of *seeds* from the target population who are keen to participate in the survey and usually socially well connected. The *seeds* are then asked to refer a pre-defined number, or ‘quota’, of contacts to the survey who form *wave* 1 of recruitment; these responders are then asked to refer *wave* 2 and so on. In this way, a sample with maximal recruitment, i.e. a full quota of new recruits in each *wave*, expands geometrically. Recruitment throughout the *waves* is driven by a primary incentive for taking part and usually a secondary incentive for recruiting others [8].

Harnessing social influence through the use of incentives gives RDS the potential to reach participants who would not normally come forward to a researcher, and the limited recruitment quota (usually three) minimises selection bias for those with large social networks [8]. This allows the characteristics of a sample to reach a steady state or ‘equilibrium’ quickly—often after just four *waves* of recruitment [8]. In addition, specific software has been developed which incorporates estimators to calculate prevalence estimates for the entire target population from data collected during the sampling process [9, 10].

However, these estimators rely on methodological assumptions, which relate to the underlying size and network structure of the target population and participant recruitment behaviour [11, 12].

Previous reviews of RDS in HIV bio-behavioural surveys [13, 14] have highlighted concerns about the quality of reporting and led, in 2015, to the publication of the Strengthening the Reporting of Observational Studies in Epidemiology for respondent-driven sampling studies (STROBE-RDS) reporting checklist [15]. This document aims to improve the quality of reporting and includes 22 items that outline how studies should report survey data collected using RDS. Importantly, it incorporates criteria that indicate sensitivity to the assumptions underlying the population estimates.

Whilst the use of RDS in HIV epidemiology has been the subject of several systematic reviews, its use in the investigation of HCV epidemiology and specifically the sensitivity of prevalence estimates to the assumptions of RDS is not described [13, 14]. The aims of this systematic review are to identify published studies documenting the use of RDS in HCV bio-behavioural surveys of PWID and describe the sensitivity of population estimates to the theoretical assumptions of RDS. To do so, the reported operational and analytical conduct of each study is compared against selected criteria from the STROBE-RDS checklist [15].

## Method

The systematic review protocol was published on the PROSPERO website under registration number CRD42015019245 prior to commencing the literature search, and the review was conducted according to the PRISMA statement [16, 17].

### Information sources and literature search

Two scoping searches were initially undertaken using MEDLINE in March 2015 with no date or language limitations. The first used the terms ‘PWID* or IDU* or Injecting drug user* AND Hepatitis C or HCV AND respondent driven sampl*’. On title and abstract review, this identified 14 potentially eligible studies and was compared to a second scoping search for the term ‘respondent driven sampl*’; this identified three additional studies suggesting the initial search was too specific.

Accordingly in the final search, MEDLINE, SCOPUS and WEB of SCIENCE online databases were searched, with no language or date limitations for the broad term ‘respondent driven sampl*’ between the 10 April 2015 to 31 December 2016*.* This was followed by a forward and backward citation search in the SCOPUS database and a manual citation search through selected papers.

Further searching was conducted through ‘grey literature’ sources including institution and key author websites, which included Respondentdrivensampling.org (Cornell University) and lisagjohnston.com. Specific search phrases in these domains varied but reflected the inclusion and exclusion criteria. Two experts with experience undertaking surveys and teaching in this field were also contacted and asked to comment on the included studies and suggest others that may meet the inclusion criteria.

### Inclusion criteria and study selection

Peer-reviewed studies written in English were included if they:

- Reported a survey in a population of PWID
- Reported the use of RDS as the sampling method
- Reported a sample prevalence or an estimated population prevalence for HCV

As HCV can remain asymptomatic and therefore undiagnosed for many decades after infection, ‘PWID’ was interpreted as anyone who had ever injected drugs [18]. Studies using mixed survey methods (for example, combined convenience sampling and RDS) and not reporting results separately were excluded. Non-English language papers were not eligible for inclusion as translation services were beyond the resources of this review; however, this was deliberately not a specific search criterion so the quantity of otherwise eligible non-English literature could be assessed.

Duplicate studies were removed from selected titles and abstracts in Mendeley reference manager. Two researchers (RB and JC), experienced in systematic review methodology, independently assessed the selected titles and abstracts for inclusion using a selection tool and resolved discrepancies by discussion with a third researcher (JP). The full papers of selected abstracts were obtained and subject to further independent review for inclusion. Where two studies reported data from the same survey and both published HCV prevalence, the study that was published first was included.

### Data extraction

Data was extracted under three headings: (1) survey overview, (2) survey outcomes and (3) reporting against selected STROBE-RDS criteria.

RB and JC extracted the data independently, and where referenced, additional papers describing the survey method in more detail were accessed and further details recorded.

## Results

### Search results

**Fig. 1:**
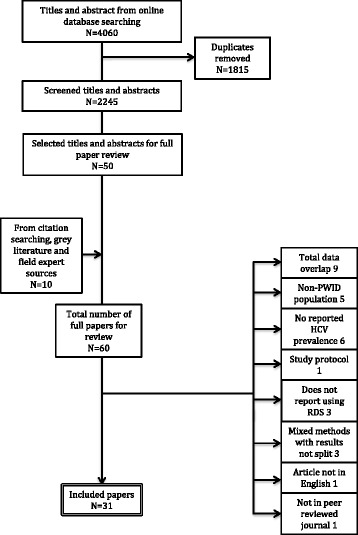
Flow diagram of studies screened and assessed for inclusion. Specific reasons for exclusion are indicated

The initial search of the online databases identified 4060 titles; of these, 1815 were duplicates leaving 2245 separate studies. Abstract and title review identified 50 studies potentially meeting the inclusion criteria. Citation, grey literature searches and expert recommendation identified a further ten studies for full paper review (Fig. 1). Sixty studies were obtained and reviewed in full. A further 29 were excluded at this stage with 31 remaining that met the inclusion criteria; Fig. 1 outlines the specific reasons for exclusion.

### Overview of included surveys

Included studies were published between 2006 and 2016 and reported either a sample or population prevalence of HCV in PWID. They included surveys from Europe, North America, Asia, the Middle East, Africa and Australasia.

Eighteen studies (58%) conducted RDS in a single target population although this varied with the largest taking place in 15 cities across India [19]. Of the studies reporting from multiple locations, two used overlapping data from the same survey [20, 21] and one study included survey sites that did not use RDS [22].

All studies clearly defined their eligibility criteria for participation and reported how the sample prevalence of HCV was obtained (Table 1). Fifteen surveys (48%) reported how participants were followed up by the research team, in most of these participants were advised to collect their testing results and were traced back to these via a ‘linked anonymous record’, i.e. the participant retained a unique identifier that connected them to their blood sample. However, two studies actually reported incentivising participants to return to collect their results [20, 21], three described a direct referral pathway from the research team to specialist services [20, 23, 24] and one of these also took the opportunity to give out harm reduction advice and, where necessary, vaccination against hepatitis A and hepatitis B [20].

**Table 1:** An overview of studies meeting the inclusion criteria

First author	Year of publication	Country	Survey duration (months)	Target populations	Target sample size per population (*deff*)	Eligibility criteria	HCV test
Abadie et al. [24]	2016	Puerto Rico	3	4		>18 years injected in last 30 days	POC antibody
Bacak et al. [55]	2013	Montenegro	4	1		18–51 years, injected in last 30 days	POC antibody
Baumbach et al. [20]^a^	2008	USA/Mexico	5	2		≥18 years, injected in last 30 days	Venipuncture antibody
Bouscaillou et al. [23]	2014	Georgia		1	193	≥18 years, injected in last 30 days	POC antibody and RNA
Burt et al. [56]	2009	USA	5	1		>18 years, injected in last 12 months	Self report
Cepeda et al. [57].	2013	Russia	23	2		≥18 years, injected last 30 days, drinks alcohol	Venipuncture antibody
Eritsyan et al. [58].	2013	Russia	2	8	300(0)	≥18 years, injected in last 30 days	Venipuncture antibody
Frost et al. [21].	2006	USA/Mexico	3	2	200	≥18 years, injected in last 30 days	Venipuncture antibody
Gelpi-Acosta et al. [38].	2011	USA	30	1	500	18–40 years, injected in last 12 months	Venipuncture antibody
Handanagic et al. [34]	2016	Croatia	4	3	350–400	>18 years, injected in last 30 days	Venipuncture antibody
Heimer et al. [28].	2014	USA		1		≥18 years, injected in last 30 days	Venipuncture antibody
Hope et al. [59].	2011	UK	3	1		≥18 years, injected in last 30 days	DBS antibody and RNA
Jarlais et al. [60]	2016	Vietnam	<1	1	600	>18 years, currently injecting	Venipuncture antibody
Johnston et al. [25]	2011	Mauritius	3	1^b^	500(2)	≥15 years, injected in last 30 days	Venipuncture antibody
Judd et al. [40].	2009	Serbia and Montenegro	2	2		≥18 years, injected in last 30 days	DBS Antibody
Lausevic et al. [36]	2015	Montenegro		1	376	≥18 years, injected in last 30 days	Venipuncture antibody
Li et al. [26]	2014	China		1	362	≥18 years, injected in last 6 months	Venipuncture antibody
Mahanta et al. [61]	2008	India	11	5	400	Males ≥18 years, injected in last 6 months	DBS antibody
Mahfoud et al. [27]	2010	Lebanon	2	1		≥15 years, injected in last 12 months	DBS antibody and RNA
Malekinejad et al. [33]	2011	USA	8	1		18–70 years, IDU in last 12 months	Self report
Mirzoyan et al. [37]	2013	Libya	5	1		≥15 years old 30 days	Venipuncture antibody
Nadol et al. [22]	2015	Vietnam	13	4	291–310(1.2)	≥18 years, injected in last 30 days	Venipuncture antibody
Paintsil et al. [41]	2009	Russia	2–7	1		≥18 years, injected in last 6 months	Venipuncture antibody
Paquette et al. [32]	2011	Australia	5	1	258(1.5)	>18 years, IDU in last 30 days	Self report
Sarna et al. [35]	2012	India	5	2	760(1.5)	Male, >16 years, IDU in last 6 months	Self report
Solomon et al. [19]	2015	India	1.5	15	1000	≥18 years, injected in last 2 years	Venipuncture antibody
Stulhofer et al. [31]	2012	Israel	3	1		18 to 56 years, injected in last 30 days	Venipuncture antibody
Tun et al. [62]	2013	Nigeria	1.5	1	400(1.4)	≥18 years, injected in last 12 months	Venipuncture antibody
Vorobjov et al. [63]	2009	Estonia		1		≥18 years, injected in last 2 months	Venipuncture antibody
Wenz et al. [29]	2016	Germany	2	8	200–400	>16 years, injected in last 12 months	DBS antibody and RNA
Zamani et al. [30]	2010	Iran	3	1	130(1.5)	≥18 years, injected in last 30 days	Venipuncture antibody

Where no information available cells left blank

*Deff* design effect, *IDU* injecting drug use, *POC* point of care test, *DBS* dry blood spot test

^a^Additional survey site excluded as reported earlier by Frost *et al.* [21]

^b^Two separate survey locations were used in Mauritius but as there was cross recruitment between sites the results were treated as a single population

Twenty studies (65%) reported the time taken to reach the final sample size, and seventeen studies (55%) documented a target sample size although of these, only seven reported the value of the design effect (*deff*) used in making the calculation (Table 1). The final sample size at each survey site was reported in most studies (97%) (mean 382; range 81–1000), and in accordance with the inclusion criteria, all the selected papers published either the sample HCV prevalence or a population prevalence estimate.

Two studies (6%) went on to use sampling data in combination with ‘service multipliers’ to calculate a total population size of PWID and therefore gave an indication of the total disease burden of HCV in the target population for the survey [25, 26].

### Sensitivity to RDS assumptions

Four studies deliberately treated their survey data as a convenience sample and did not report any intention to calculate population estimates. The remaining 27 studies either calculated or reported the intention to calculate population prevalence estimates, and these studies were therefore constrained by the theoretical assumptions underlying RDS.

Table 2 gives an overview of reporting in these studies against selected STROBE-RDS criteria that give an indication about sensitivity and adherence to these assumptions. The following section describes how these 27 studies reported against these criteria and where there was evidence that the assumptions were not met.

**Table 2:** STROBE-RDS criteria indicating adherence or sensitivity to RDS assumptions in included studies reporting or intending to report a population prevalence estimate for HCV

Study	Formative research undertaken	Research venue	Incentive primary/secondary	Number of *seeds* per site	Max recruitment *waves*	*Seed* data in analysis	Software used
Abadie et al. [24]		NSP	£/£	2			RDSAT/RDS-A
Bacak et al. [55]		HIV counselling office	£/£				RDSAT
Baumbach et al. [20]		NGO clinic	£/£	5			RDSAT
Bouscaillou et al. [23]		Drug support drop in centre	£/		9		RDSAT
Frost et al. [21]		Mobile bus and NGO clinic	£/£	12	8	Excluded	RDSAT
Gelpi-Acosta et al. [38]	Field observation and interviews [64]	Field office or mobile van	£/£			Excluded	RDSAT
Handanagic et al. [34]			Food coupon/food coupon	13.7			RDS-A
Heimer et al. [28]				82			RDSAT
Hope et al. [59]			£/£		17		RDSAT
Jarlais et al. [60]			£/£	12			RDSAT
Johnston et al. [25]	Interviews with local officials	NGO centre, rented space	£/£	6	13	Included	RDSAT
Judd et al. [40]		Shopping mall and NGO centre	£/£	3			RDSAT
Lausevic et al. [36]			£/	5	10	Excluded	RDS-A
Li et al. [26]	Informal stakeholder meetings	Drop in centre	£/£	5	11		RDSAT
Mahanta et al. [61]				3			RDSAT
Mahfoud et al. [27]		NGO centres	£/£				RDSAT
Malekinejad et al. [33]	Key informant interviews, focus group		£/£	16	27		RDSAT
Mirzoyan et al. [37]			£/£	7	10		RDSAT
Nadol et al. [22]	Formal study		£/£		8		RDS-A
Paintsil et al. [41]			Gifts/gifts	23		Excluded	STATA
Paquette et al. [32]		NSP	£/£	5	16		RDSAT
Sarna et al. [35]		NGO centre	£/£	4.5		Excluded	RDSAT
Solomon et al. [19]	Field observation	Drop in centre	/£	2.1	50		RDSAT
Stulhofer et al. [31]	Interviews with local officials		£/£	7	12	Excluded	RDSAT
Tun et al. [62]	Interviews with PWIDs	NGO centre	£/£	7			RDSAT
Wenz et al. [29]	Interviews with key stakeholders	Drop in centre	£/£	7-19	20	Included	RDSAT
Zamani et al. [30]	Interviews with local staff and focus groups with PWIDs	Drop in centre	Gift/none	10	8		RDSAT

Where no information available cells left blank. For RDSAT, see reference [9], and for RDS-A, see reference [10]

*RDS* respondent-driven sampling, *NGO* non-governmental organisation, *NSP* needle syringe programme, *BBV* blood borne virus, *STI* sexually transmitted infection, *£* financial incentive given

#### Assumption 1: participant social networks are linked into a single component

There were indications given in three studies (11%) that the underlying network structure adversely affected recruitment [20, 27, 28] and from a single study that clustering within the network affected the validity of population prevalence estimates [29]. These studies did not describe formative research to explore the structure of the social network in advance of the survey, but this was described in nine other studies. Among these, there was variation in the scale and methods used; some studies reported the use of informal interviews with local stakeholders, whilst others described focus groups, qualitative interviews and ethnography or cited a published preliminary study. Only one study specifically described how this formative work was used to optimise recruitment from all parts of the network [30].

#### Assumption 2: recruiters do not pass coupons to strangers and ties are reciprocal

Two studies (7%) reported a number of participants being recruited to the survey by strangers, but neither described how these participants were handled in the analysis [30, 31]. Overall, sixteen studies (59%) reported the recording of the relationship between the recruiter and recruit; however, only one precisely defined the question that was used to assess this [32].

#### Assumption 3: estimates are independent of seed characteristics

Eight studies (30%) reported the purposive selection of *seeds* through ethnography or via consultation with key stakeholders in the field. Nineteen studies (70%) described the number of *seeds* used to initiate recruitment (range 2 to 82) although only two met the STROBE-RDS checklist by describing clearly how many *seeds* were added to boost recruitment after the survey had started [33, 34]. The data from one survey could not be used to calculate a population prevalence for HCV because too many *seeds* had been needed to reach the target sample size [28].

The recruitment quota, or number of coupons given to each *seed*, was reported in all studies and ranged from 2 to 4, but the number of recruitment *waves* per *seed* was poorly described with only three studies including diagrammatic recruitment ‘trees’ within the main text [21, 29, 33]. However, 15 studies (56%) reported the number of *waves* achieved in the longest recruitment chain (range 5–50), and another reported a median chain length across 15 survey sites [19]. Seven studies (26%) reported measuring ‘sampling equilibrium’ after a certain number of recruitment *waves* for key criteria to indicate independence of the sample from *seed* characteristics and one used this as the point to stop sampling [26].

How *seed* data was handled in the analysis was not explicitly reported in most studies although six (22%) did describe deliberately excluding *seed* data from population prevalence estimates whereas two (7%) specifically documented its inclusion [25, 29].

#### Assumption 4: recruiters pass coupons randomly to eligible network members and these individuals are equally likely to participate

One study clearly described how participants were trained to recruit social network members to the survey [33], but there were concerns expressed in a number of studies about non-random recruitment. Eight studies (30%) reported difficulty recruiting female participants despite, in one, the deliberate use of female seeds [23]. One study considered whether this was a true representation of the underlying population structure [35], but three expressed concern about ‘response bias’ attributed to cultural barriers within the target population [23, 36, 37]. Other studies expressed concerns about the non-recruitment of participants from particular ethnic backgrounds [32], socio-economic groups [28] or geographical areas [32, 33]. To test recruitment bias, three studies (11%) reported measuring homophily for selected characteristics between recruits and recruiters [24, 29, 34] and in one homophily between persons with a known HCV-positive status was recorded [24].

Sixteen studies (59%) described the venue used for the survey, and one reported concerns that the venue may have influenced participation [34]. The incentives used for recruiting others to the survey were also described in 21 studies (78%), and 19 of these described a financial primary and secondary incentive, the value for which ranged from $50 and $20, respectively, in the USA [38] and $1 and $0.8 in India [19, 35]. Where reported, the remaining surveys used gifts or food coupons [30, 34, 39]. A single survey expressed concern that the financial incentive may have led to bias towards poorer PWID and one did not use a secondary incentive for this reason [30]. Another study was concerned that the offer (as part of participation) of being linked directly to HCV care may have encouraged a disproportionate number of PWID with HCV to attend [23].

#### Assumption 5: participants only take part once and are eligible members of the target population

A single study described participants attempting to attend more than once and non-eligible individuals trying to take part [40]. The method used to screen survey participants for eligibility (i.e. proof they had injected drugs) was recorded in 15 studies (56%), but only four described how repeat attenders were identified. Of these, a single study recorded identifiers such as tattoos or anthropometric measurements [41] and one used finger print records [19].

#### Assumption 6: participants accurately report their degree size

Fifteen studies (56%) reported recording the *degree* size for each recruit, and of these, three precisely described the question or questions used to define this [20, 29, 33]. No studies reported testing the sensitivity of prevalence estimates against variations in degree size.

#### Assumption 7: sampling occurs with replacement

The majority (85%) of included studies used a version of RDSAT software [9] to calculate prevalence estimates. RDSAT incorporates an estimator that is constrained by this assumption [11, 42, 43]; however, just a single study measured how this may have affected the HCV prevalence estimate by comparing it against an estimate calculated with a successive sampling estimator [24].

#### Assumption 8: an estimate of total target population size is known in advance of the survey

Four studies (15%) used a successive sampling estimator integrated within RDSanalyst software [10] to calculate population estimates and therefore needed a target population size estimate to make the calculation. Two specifically reported the use of such an estimate and referenced its source [22, 34].

## Discussion

The studies included in this review used RDS to recruit over 25,000 PWID to bio-behavioural surveys across five continents. The studies were consistent in documenting the use of standard RDS methods including recruitment coupons, recruitment quotas and incentives to facilitate the coupon exchange but varied considerably in scale, duration and operational conduct.

The quality of reporting against the STROBE-RDS criteria, in some instances, made an assessment about the sensitivity of survey results to the underlying assumptions of RDS difficult. The incomplete reporting of the sampling method in surveys using RDS has been described before [13, 14] and is not surprising here given that the STROBE-RDS checklist was published after most of the included studies [15]. Nevertheless, from what was reported, there were indications that the assumptions were not met in some studies, and in two cases, this led to study authors being unable to use survey data to calculate a population prevalence estimate. This is consistent with reports elsewhere which describe recruitment via non-reciprocal relationships [44], inaccurate degree size reporting [45], biased recruitment according to ethnicity [46] and limited recruitment due to disparate social networks within the target population [47].

The collective understanding of the implications of not meeting the assumptions of RDS has advanced in recent years through literature ‘testing the assumptions’ [45, 48–51]. Simulation studies have reported the scale of biases associated with *seeds*, recruitment *waves*, high recruitment homophily and sampling *without* replacement [50], whilst work based on real-world surveys has demonstrated the bias associated with inaccurate reporting of degree size [45]. This has led to the evolution of the original RDS estimator [11, 42, 52], new estimators based on successive sampling and *ego* network data [53, 54], and development of RDS technical procedure—an iterative temporal transformation that may account for some of the variation seen in the included studies.

Specifically, this has led to development in how to accurately ascertain degree size, how to handle *seed* data in the analysis [50] (a contrast with earlier literature [11]), how to measure sample independence from *seed* characteristics using convergence rather than equilibrium [12], and the use of ego network data to assess recruitment bias (65).

This systematic review is the first to describe the use of RDS in HCV epidemiology and explore sensitivity to the methodological assumptions underlying RDS in these studies. In so doing, it draws attention to reporting criteria for surveys using RDS and highlights recent technical developments. However, it also has areas of potential bias, for example, the search strategy, by including only peer-reviewed publications, excluded survey data within grey literature such as public health reports. This may have led to bias towards the more successful, robustly designed surveys that have a higher chance of publication. In so doing, this review may have overestimated the quality of reporting relating to the assumptions of RDS and underestimated sensitivity to these assumptions.

## Conclusion

RDS can improve our understanding of HCV epidemiology in PWID and therefore has the potential to make an important contribution to the global elimination strategy for HCV. This robust systematic review included 31 studies and showed that operational procedures varied between studies and were frequently incompletely reported. There were also widespread indications of sensitivity to the methodological assumptions of RDS that, in some studies, prevented the estimation of HCV population prevalence. Future surveys using RDS to explore the epidemiology of HCV within PWID should convey sensitivity to the assumptions by reporting in accordance with the STROBE-RDS checklist and should consider using recent advances in the procedural and analytical methods of RDS.

## Abbreviations

Deff: Design effect
HCV: Hepatitis C
PWID: People who inject drugs
RDS: Respondent-driven sampling
